# A topical microemulsion for the prevention of allergic rhinitis symptoms: results of a randomized, controlled, double-blind, parallel group, multicentre, multinational clinical trial (Nares study)

**DOI:** 10.1186/1710-1492-9-32

**Published:** 2013-08-27

**Authors:** Pedro Ojeda, Núria Piqué, Alicia Alonso, Julio Delgado, Francisco Feo, Juan Manuel Igea, Ana Navarro, José María Olaguibel, Javier Subiza, Carles Nieto, Morgan Andersson

**Affiliations:** 1 Clínica de Asma y Alergia Dres, Ojeda, Oquendo, 23, 20006 Madrid, Spain; 2 Department of Microbiology and Parasitology, Pharmacy Faculty, Universitat de Barcelona, Av. Joan XXIII, 31, 08028 Barcelona, Spain; 3 Clínica Paracelso Médico Quirúrgica, General Ruiz, 4, 47004 Valladolid, Spain; 4 Department of Allergy, University Hospital Virgen Macarena, Av. Dr. Fedriani, 3, 41071 Seville, Spain; 5 Department of Allergy, University Hospital of Ciudad Real, Obispo Rafael Rorija, 13005 Ciudad Real, Spain; 6 Alergoasma Clinic, Pinto, 2-18, 37001 Salamanca, Spain; 7 Hospital Nuestra Señora de Balme, Ambrosio de la Cuesta, 11, Seville, Spain; 8 Allergy Department, Complejo Hospitalario de Navarra, Irunlarrea, 3, 31008 Pamplona, Spain; 9 Clínica Subiza de Asma y Alergia, General Pardinas, 116, 28006 Madrid, Spain; 10 Reig Jofré Group, S.A., Gran Capità, 6, 08970 Sant Joan Despí, Barcelona, Spain; 11 Department of Otorhinolaryngology, Head & Neck Surgery, Skåne University Hospital, Ole Römers väg, 3, 22363 Lund, Sweden

**Keywords:** Allergic rhinitis, Rhinoconjunctivitis, Allergen avoidance, Barrier measures, Corticosteroids use, Nasal symptoms, Natural pollen exposure, Quality of life, Efficacy, Safety

## Abstract

**Background:**

Since barrier protection measures to avoid contact with allergens are being increasingly developed, we assessed the clinical efficacy and tolerability of a topical nasal microemulsion made of glycerol esters in patients with allergic rhinitis.

**Methods:**

Randomized, controlled, double-blind, parallel group, multicentre, multinational clinical trial in which adult patients with allergic rhinitis or rhinoconjunctivitis due to sensitization to birch, grass or olive tree pollens received treatment with topical microemulsion or placebo during the pollen seasons. Efficacy variables included scores in the mini-RQLQ questionnaire, number and severity of nasal, ocular and lung signs and symptoms, need for symptomatic medications and patients’ satisfaction with treatment. Adverse events were also recorded.

**Results:**

Demographic characteristics were homogeneous between groups and mini-RQLQ scores did not differ significantly at baseline (visit 1). From symptoms recorded in the diary cards, the ME group showed statistically significant better scores for nasal congestion (0.72 vs. 1.01; p = 0.017) and mean total nasal symptoms (0.7 vs. 0.9; p = 0.045). At visit 2 (pollen season), lower values were observed in the mini-RQLQ in the ME group, although there were no statistically significant differences between groups in both full analysis set (FAS) and patients completing treatment (PPS) populations. The results obtained in the nasal symptoms domain of the mini-RQLQ at visit 2 showed the highest difference (−0.43; 95% CI: -0.88 to 0.02) for the ME group in the FAS population. The topical microemulsion was safe and well tolerated and no major discomforts were observed. Satisfaction rating with the treatment was similar between the groups.

**Conclusions:**

The topical application of the microemulsion is a feasible and safe therapy in the prevention of allergic symptoms, particularly nasal congestion.

**Trial registration:**

ClinicalTrials.gov Identifier: NCT01478425

## Background

In the management of allergy, there are four general principles: patient education, avoidance of allergens/triggering factors, use of appropriate pharmacotherapy and immunotherapy [1,2]. Importantly, allergen avoidance should be indicated when possible and should be an integral part of the management strategy, according to the clinical and practical recommendations of ARIA guidelines for the management of allergic rhinitis [2,3].

With regard to allergen avoidance or modification of allergen exposure, barrier protection measures for avoiding contact with allergens, such as nose filters or nasally applied cellulose powders, are being increasingly developed and evaluated in patients with allergic rhinitis [4]. In this context, a topical microemulsion made of glycerol esters for topical application in the nose has been developed, with the aim of conferring a protective effect in patients with allergic rhinitis. Its mechanism of action consists of creating a lipid coating that spreads over the surface of the nasal mucosa. Acting this way, the protective effect is achieved by creating a lipid barrier that prevents allergens being deposited onto the nasal mucosa and reaching the specific IgE of the mucosal immune system cells and engulfing allergens already present in the nasal mucosa. As such, the allergic reaction will be blocked by the application of the microemulsion at the very beginning of the allergic cascade, in contrast to commonly used symptomatic medications such as antihistamines or corticosteroids, which act at the end of this cascade.

Previous studies have shown that the application of the topical microemulsion caused a reduction of nasal symptoms scores during one week of treatment in symptomatic patients with perennial allergic rhinitis due to house dust mites [5]. In a single-blind, placebo-controlled, crossover study, a reduction in nasal symptoms scores and a drop in a2-macroglobulin levels in nasal lavage fluid, indicative of attenuation of inflammation, were also observed after challenge with pollen allergen in patients with allergic rhinitis [6].

In this study, which included patients with seasonal allergic rhinitis, the effect of a topical microemulsion was examined in a natural allergen exposure setting (during birch, grass, and olive tree pollen seasons). The intervention was given according to a placebo-controlled, double-blind, randomized, parallel group design in nine study centres in Spain and Sweden. Rhinitis-specific quality of life and symptoms characteristic of seasonal allergic rhinitis were monitored throughout the pollen season. We report on the symptom-reducing effects of the intervention.

## Methods

The study protocol was approved by the Ethics Committee of Human Experimentation in Spain and Sweden and procedures were in accordance with the ethical standards laid down in the Declaration of Helsinki, as revised in the year 2000. Written informed consent was obtained from all subjects. Patients were recruited in one Swedish centre (n = 22) and in 8 Spanish centres (n = 88), with a varied geographical distribution in order to cover the different pollination patterns.

This randomized, controlled, double-blind, parallel group, multicentre, multinational clinical trial (8 centres in Spain and 1 in Sweden) was performed to evaluate the efficacy and tolerability of the topical microemulsion (ME group) compared to a normal saline solution (SS group) in adult patients with moderate to severe allergic rhinitis or rhinoconjunctivitis due to sensitization to grass, birch, or olive tree pollens. The diagnosis was made according to the investigators’ judgment based on the clinical picture correlated with positive skin prick tests (wheal diameter ≥ 3 mm) and/or specific IgE (titer ≥ 0.35 kU/L) for the allergenic extracts under consideration and routinely used by the investigators in their clinical practice. Potential participants were excluded if they had asthma of any origin, seasonal rhinitis with negative allergy testing, had received allergen-specific immunotherapy within the previous 36 months, or were on systemic corticosteroids or other immunosuppressant or immunomodulatory drugs.

The patients were randomly assigned to receive the microemulsion (ME group) or the saline solution (SS group). The demographic characteristics are shown in Table 1. The composition of the topical microemulsion was glycerol monooleate, propylene glycol, polyethylene glycol 400, sesame oil, polysorbate 80, sodium chloride 0.9%, menthol, eucalyptus oil and water, whereas the placebo was composed of sodium chloride 0.9%, menthol, eucalyptus oil and water at a neutral pH. Both products were manufactured by Reig Jofré (Reig Jofré Group S.A., Barcelona, Spain), according to international Good Manufacturing Practices. Treatment (one puff of either product b.i.d. per nostril, in the early morning and at midday) was administered throughout the expected dates of the pollen seasons for the pollens considered in this study. The pollen periods were defined according to the pollen counts provided by local agencies or networks, from the start of significant pollen counts until the end of significant pollen counts. Three visits were performed: just before the beginning (visit 1), in the middle (visit 2) and at the end of the pollen season (visit 3).

**Table 1:** Baseline demographic characteristics of the study subjects

		**Statistic variable**	**ME group**	**SS group**
Gender (M / F)		n (%)	28 (58.8) / 25 (47.1)	25 (48.0) / 27 (51.9)
Age (years)		Mean (SD)	32.6 (9.9)	34.9 (11.5)
		Range	18.0 - 57.0	18.0 – 69.0
Duration of rhinitis (years)		Median (IQR)	11.0 (9.0)	10.0 (13.0)
		Range	1.0 - 33.0	1.0 - 47.0
Sensitization profile		% of subjects testing positive at SPT		
Grass pollen			84.6	92.4
Olive tree pollen			46.1	52.8
Birch tree pollen			25.0	22.6

***SPT*** Skin Prick Test, ***SD*** Standard Deviation, ***IQR*** Interquartile range.

At each visit, the investigators performed a physical examination on each subject indicating the presence or absence of the following symptoms: rhinorrhea, nasal edema, nasal mucosa pallor, nasal crusts, eye redness, epiphora, conjunctival secretion, prolonged expiration, rhonchi or wheezing, and tachypnea. The participants completed a mini-RQLQ questionnaire (7, 8). At visit 1, the patients were provided with a daily symptoms and rescue medication consumption diary to be completed every day, at the end of the day, from visit 1 to visit 3. The severity of nasal symptoms (itching, runny nose, sneezing, and nasal congestion) and ocular symptoms (itching, redness, and tearing) was scored according to the following scale: 0 = no symptoms; 1 = mild symptoms; 2 = moderate symptoms; 3 = severe symptoms, based on the patient’s opinion. The patients were allowed to take topical antihistamines or corticosteroids, as well as commercially available oral antihistamines on an on-demand basis and according to the prescriptions made by the investigators. The brands as well as the number of times used per day were recorded on the dairy. Adverse events were recorded at visits 2 and 3, and a questionnaire on the patient’s satisfaction with the treatment was completed at visit 3 (very satisfied; neither satisfied nor dissatisfied; dissatisfied; very dissatisfied).

The primary efficacy variable was the overall allergic rhinitis-related quality of life calculated by the least-squares mean of global scores in the self-administered mini-RQLQ questionnaire at visit 2 in the FAS population (randomized patients meeting all selection criteria and having a mini-RQLQ value at baseline). Secondary efficacy variables included: mini-RQLQ scores at visits 2 and 3 in the FAS (full analysis set) and PPS populations (FAS patients who completed the study treatment, did not take prohibited medications and had a value for the primary variable); number of nasal, ocular and lung signs (from the physical examination); severity of nasal and ocular symptoms, measured as the mean of nasal symptoms and of ocular symptoms (the sum of nasal symptoms divided by 4; the sum of ocular symptoms divided by 3; range: 0–3); need for symptomatic medications; adherence (based on the medication returned by subjects); and the patient’s satisfaction with treatment.

Descriptive statistics were used for each outcome and intra and inter-group comparisons were made. Two-sided p-values were obtained and statistically significant results were declared if p < 0.05. The 95% confidence intervals were computed for the treatment effect, using either least-squares mean difference or Hodges-Lehmann estimators. ANCOVA analysis was considered in appropriate cases and a Mantel-Haenszel Chi-square test was also performed. The statistical significance level was calculated at two-sided and one-sided p values. Homogeneity of association across centres was assessed by the Breslow-Day test.

## Results

**Figure 1:**
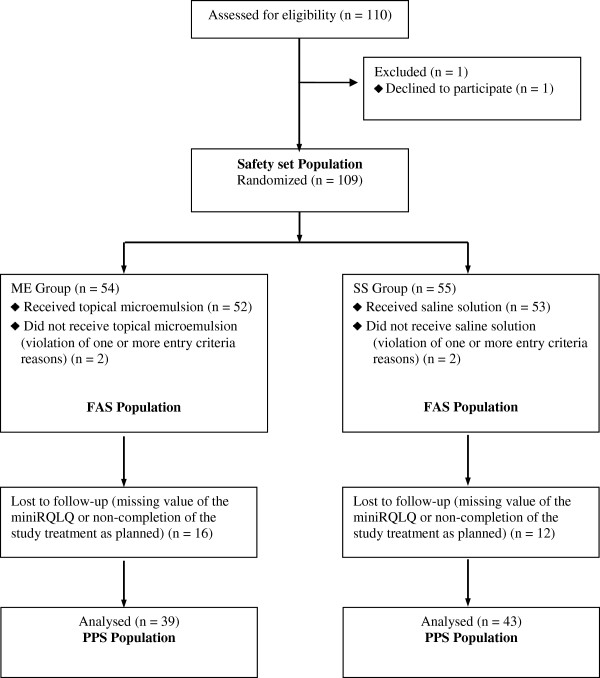
**Flow diagram of the clinical trial progress.** Safety Set (SS): Randomized patients who took at least one dose of the study treatment; Full Analysis Set (FAS): randomized patients who met all selection criteria and had a value of the mini-RQLQ at baseline (visit 1); Per Protocol Set (PPS): FAS patients who completed the study treatment, did not take prohibited medications and who had a value for the primary variable (mini-RQLQ at visit 2).

A total of 110 patients were included in the study database (n = 55 in each treatment group). Figure 1 shows the study retention data.

The study duration (period between visits 1 and 3) ranged from 29 to 133 days, depending on the duration of the pollen season in the different geographical areas. Demographic characteristics were homogeneous between groups, as were the duration of allergic rhinitis and the sensitization profiles (Table 1). Rhinorrhea, edema of the nasal mucosa, nasal mucosa pallor and crusts, eye redness and epiphora were seldom present at baseline in both groups and these findings significantly increased in both groups at visit 2.

**Figure 2:**
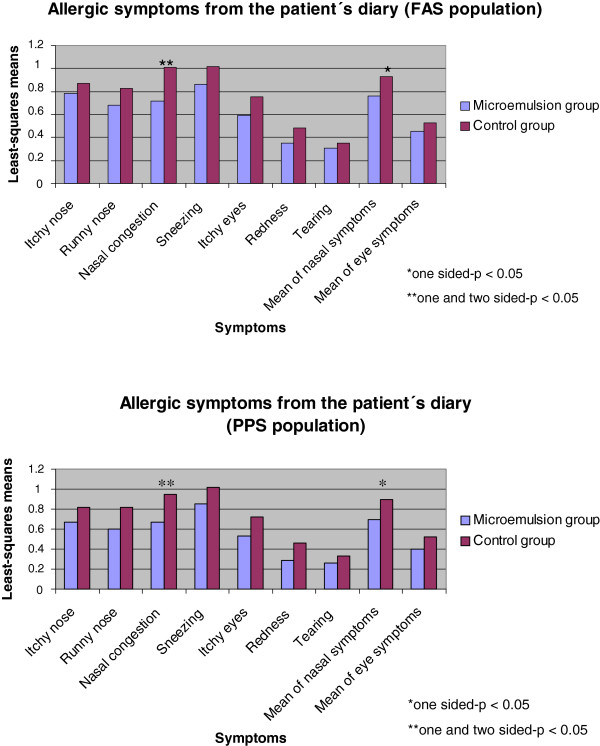
**Allergic symptoms.** Allergic symptoms recorded in the patient’s diary as daily means (FAS and PPS populations).

In general, efficacy results were favorable for the microemulsion. The least-squares means for the allergic symptoms recorded in the patients’ diary cards were always lower in the active group than in the control group, in both FAS (full analysis set) and PPS (per protocol set) populations. In this respect, statistically significant differences were detected in the FAS population for nasal congestion (p = 0.011), while the difference in mean scores for nasal symptoms did not reach statistical significance (p = 0.068). In the PPS population, significant differences were found for nasal congestion (p = 0.017) and mean nasal symptoms (one-sided p = 0.044) (Figure 2).

**Figure 3:**
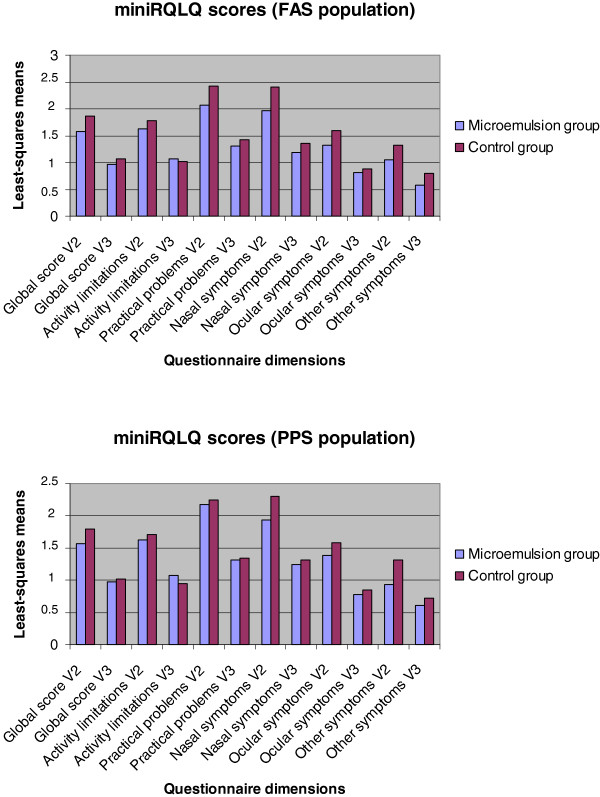
**Patient’s quality of life.** Results of quality of life, global and in the different dimensions of the mini-RQLQ questionnaire (FAS and PPS population).

Lower global scores in the mini-RQLQ at visit 2 in the FAS population, which was the primary efficacy variable, were also observed in the group of patients treated with the microemulsion, indicating a better overall quality of life (Figure 3), although without reaching statistically significant differences between the groups.

In both populations and at both visits, least-squares means of the global and single dimension scores in the mini-RQLQ were also lower in the active than in the control group (except for activity limitations at visit 3) (Figure 3).

Of note, results obtained in the nasal symptoms domain of the mini-RQLQ at visit 2 showed the highest difference (−0.43; 95% CI: -0.88 to 0.02, p = 0.059) in the FAS population. The scores obtained at visit 2 were higher than at visit 3, thus indicating a better quality of life at the end of the study, which corresponded to the end of the pollen season and was in agreement with the reported incidence of allergic symptoms (Figure 3).

Correspondingly, nasal signs recorded at the physical examination were more frequent in the control group at both visits, with RR values below 1. Eye signs were also more frequent at visit 3 in the control group (with RR = 0.42) but more frequent in the active group at visit 2 (RR = 1.45). No patients of any group presented lung signs at visit 2 and only one patient in the active group presented lung signs at visit 3.

The distribution of satisfaction scores was similar in both treatment groups (with no statistically significant differences), thus indicating no major discomfort from the application of the microemulsion. Importantly, proportions of patients taking corticosteroids were higher in the control group than in the group treated with the microemulsion, although differences were not statistically significant (RR > 1.19; 95%CI 0.67-2.11; p = 0.538). Based on the medication returned by subjects, the percentage of adherence to treatment was high (93% in the active and 92% in the control group).

The application of the topical microemulsion was safe and well tolerated, with only 3 events considered as definitely related to the microemulsion treatment (intense nasal and palatal itching/nasal itching and rhinorrhea/nasal irritation) and 8 events considered as possibly related (sneezing, pharyngeal discomfort, pharyngeal itching and bitterness, nasal itching and rhinorrhea). Only one event in the active group resulted in temporary discontinuation of the study treatment and two events in the control group resulted in definitive withdrawal. Importantly, during the study, no serious adverse events occurred and most adverse events reported in both groups were related with the allergic rhinitis pathology, in the form of nasal and respiratory symptoms.

## Discussion

In this study, which included patients with allergic rhinitis caused by birch, grass, and olive pollen allergen, we showed that topical/intranasal administration of small volumes of a microemulsion twice daily during the pollen season reduces overall nasal symptoms compared with placebo (i.e. isotonic saline). This observation is of relevance in terms of how allergen avoidance can be achieved and how microemulsions may be used as a treatment for seasonal allergic rhinitis.

This randomized, controlled, double-blind, parallel study was performed according to the international recommendations for the evaluation of products for allergic rhinitis [3] and with a similar design to other studies evaluating allergen avoidance strategies [4,6]. Quality of life is an important treatment efficacy marker in patients with allergic diseases [7] and in our study, the effect of the microemulsion was assessed using the validated and reliable mini-RQLQ questionnaire [8], commonly used in patients with rhinitis and/or rhinoconjunctivitis [7,8].

Symptoms of allergic rhinitis recorded throughout the pollen season in the placebo group indicated that intensity was mild, reaching a score of 0.90 on a scale from 0 to 3 for total nasal symptoms. In patients who received the microemulsion intervention, least squares means of all variables derived from symptoms were nevertheless always lower compared with placebo, and this pattern reached statistical significance for total nasal symptoms. Accordingly, we suggest that topical/intranasal administration of small volumes of a microemulsion may be viewed as a preventive measure or treatment option in seasonal allergic rhinitis. This conclusion may be strengthened by the fact that the present comparator, i.e. isotonic saline, may not be a true placebo since isotonic (and hypertonic) saline may be effective in allergic rhinitis. For example, Garavello et al. showed that nasal administration of isotonic saline reduced nasal symptoms in children with seasonal allergic rhinitis [9]. The possibility that saline may exert such an effect, in combination with the significant difference between the microemulsion and isotonic saline in this study, underscores that the microemulsion is effective in seasonal allergic rhinitis.

In contrast to our previous study in seasonal allergic rhinitis in which a pool-device was used for experimental administration of a large volume of microemulsion [5], this study involved repeated administration of a small volume (50 μl) using a spray-like device. Symptoms were recorded in the evening, i.e. at time points up to 6–8 hours after dosing. The results therefore suggest that the microemulsion, given in small doses, was effective for many hours. Similarly, an extended effect was also suggested in our previous study of patients with perennial allergic rhinitis who received low-dose microemulsion intervention [6]. Microemulsions containing glycerol monooleate are known to be bioadhesive and are probably not cleared by mucociliary activity as rapidly as, for example, suspensions [10]. This may explain why a small volume of microemulsion is sufficient for reducing symptoms in seasonal allergic rhinitis. However, further studies involving somewhat larger volumes and more frequent dosing may be considered to see if greater symptom-reducing effects can be achieved. Also, such studies should include comparisons with established treatments for allergic rhinitis including antihistamines and topical corticosteroids.

The findings in this study are in agreement with our previous observations on symptom-relieving effects in allergic rhinitis and support the hypothesis that the microemulsion reduces the interaction between the allergen and the mucosa in a non-specific fashion [5,6]. Accordingly, we suggest that microemulsions may be efficacious for allergic rhinitis regardless of the type of allergen producing the nasal symptoms. Indeed, interventions aiming at avoiding allergen exposure or decreasing allergen exposure in general have recently received attention. A gel-forming cellulose powder was shown to reduce rescue medication use in a seasonal allergen exposure setting [4,11,12]. Furthermore, symptoms were reduced by cellulose powder in an experimental challenge model involving dust mite allergens [13]. Taken together, the above concepts highlight the possibility that devices aiming at physically protecting the nasal mucosa against allergens may be valid treatment options in allergic rhinitis. This may be viewed as being in line with current recommendations of allergen avoidance in allergic rhinitis [3].

## Conclusions

We conclude that the topical application of the microemulsion reduces symptoms of seasonal rhinitis in a natural allergen exposure setting. Our finding suggests that topical microemulsions may be a useful option for avoiding mucosal exposure to allergens in this pathological condition. We suggest that microemulsions may be viewed as a means of reducing mucosal exposure to harmful inhaled factors in general.

## Abbreviations

ANCOVA: Analysis of covariance; ARIA: Allergic Rhinitis and its Impact on Asthma; b.i.d: Twice a day; FAS: Full analysis set; IgE: Immunoglobulin E; ME: Microemulsion; PPS: Per protocol set; RQLQ: Rhinoconjunctivitis quality of life questionnaire; RR: Relative risk; SS: Saline solution.

## Competing interests

Pedro Ojeda received honoraria from Reig Jofré for the design and study direction. Carlos Nieto is Medical Director of Reig Jofré. Morgan Andersson received financial support from Reig Jofré for the conduction of the study. Morgan Andersson holds patents pertinent to the microemulsion used in this study. Núria Piqué received honoraria from Reig Jofré to write the article.

## Authors’ contributions

Study conception and design: PO and MA. Study conduct: PO, MA, AA, JD, FF, JMI, AN, JMO, JS. Data analysis: PO. Contribution of reagents/materials/analysis tools: PO, CN. Article writing: PO, NP, MA. All authors read and approved the final manuscript.
